# Predictors of futile recanalization in basilar artery occlusion patients undergoing endovascular treatment: a post hoc analysis of the ATTENTION trial

**DOI:** 10.3389/fneur.2023.1308036

**Published:** 2023-12-18

**Authors:** Tingyu Yi, Kai Li, Xiao-hui Lin, Ding-lai Lin, Yan-Min Wu, Zhi-nan Pan, Xiu-fen Zheng, Rong-cheng Chen, Guoyong Zeng, Wen-huo Chen

**Affiliations:** ^1^Department of Neurointervention, Zhangzhou Affiliated Hospital of Fujian Medical University, Zhangzhou, China; ^2^Department of Neurology, Heze Municipal Hospital, Heze, China; ^3^Department of Neurology, Ganzhou People’s Hospital, Ganzhou, China

**Keywords:** basilar artery occlusion, endovascular treatment, futile recanalization, prognosis, diabetes mellitus

## Abstract

**Background:**

Few studies have focused on factors associated with futile recanalization in patients with an acute basilar artery occlusion (BAO) that was treated with modern endovascular therapy (EVT). The aim of this study was to explore the factors associated with futile recanalization in patients with an acute BAO presented within 12 h.

**Methods:**

This is a post-hoc analysis of the ATTENTION trial (The Trial of Endovascular Treatment of Acute Basilar-Artery Occlusion, ClinicalTrials.gov, number NCT 04751708). Demographics, clinical characteristics, acute stroke workflow interval times, and imaging characteristics were compared between the futile recanalization and favorable recanalization groups. The favorable outcome was defined as a modified Rankin scale (mRS) score of 0–3 at 90 days, successful reperfusion was defined as thrombolysis in cerebral infarction (TICI) 2b and 3 on the final angiogram, and futile recanalization was defined as failure to achieve a favorable outcome despite successful reperfusion. A multivariate analysis was performed to identify the predictors of futile recanalization.

**Results:**

In total, 185 patients were included in the final analysis: 89 (48.1%) patients had futile recanalization and 96 (51.9%) patients had favorable recanalization. In the multivariable logistic regression analysis, older age (OR 1.04, 95% CI 1.01 to 1.08, *p* = 0.01) and diabetes mellitus (OR 3.35, 95% CI 1.40 to 8.01, *p* = 0.007) were independent predictors of futile recanalization.

**Conclusion:**

Futile recanalization occurred in nearly half of patients with acute BAO following endovascular treatment. Old age and diabetes mellitus were identified as independent predictors of futile recanalization after endovascular therapy for acute BAO.

## 1 Introduction

Stroke caused by acute basilar artery occlusion (BAO) is associated with high morbidity and mortality (1, 2). Endovascular therapy (EVT) can quickly open the occluded artery and improve the prognosis of stroke patients with intracranial large artery occlusion (LVO) and is the standard treatment for LVO in the anterior circulation (3). Recently, two breakthrough trials showed the clinical benefit of EVT for BAO (4, 5). According to the results of the ATTENTION trial (the Trial of Endovascular Treatment of Acute Basilar-Artery Occlusion, ClinicalTrials. gov, number NCT 04751708) (4), the proportion of patients with successful reperfusion (modified thrombolysis in cerebral infarction (mTICI) ≥2b) was 93.0%. However, the rate of a favorable outcome (defined as a modified Rankin scale (mRS) score of 0–3) was only 46% (4), indicating that approximately 50% of patients failed to achieve a favorable outcome despite successful recanalization, and this phenomenon was defined as futile recanalization. The risk factors and incidence of futile recanalization in patients with ischemic stroke caused by BAO undergoing EVT have yet to be identified. Thus, in this study, we analyzed data from the ATTENTION trial to identify the incidence and predictive factors of futile recanalization.

## 2 Methods

The ATTENTION trial was an investigator-initiated, multicenter, prospective, randomized, open-label trial that investigated the outcomes of EVT in Chinese stroke patients with basilar artery occlusion (BAO). The study methods and patient eligibility criteria have been reported previously (4). Patients who were not followed up, did not undergo catheter angiography, or had failed recanalization (mTICI <2b) were excluded from this subgroup analysis. In order to minimize the impact of the premorbid state, patients with a premorbid mRS score of ≥1 were also excluded from this study.

The baseline information included age, sex, baseline National Institutes of Health Stroke Scale (NIHSS) score, history of hypertension, dyslipidemia, diabetes, smoking, atrial fibrillation, previous stroke/transient ischemic attack (TIA), and coronary artery disease. The cause of stroke included cardioembolic, large-artery atherosclerosis, undetermined causes, or other determined causes. The symptom onset to presentation time, reperfusion time, whether intravenous thrombolysis was performed, time of endovascular procedure initiation, and recanalization time were also recorded.

All patients underwent head CT on admission; the baseline infarct range was assessed by posterior circulation using the Alberta Stroke Program Early CT Score (PC-ASPECTS) with non-contrast CT; and the location of the intracranial artery occlusion was identified on digital subtraction angiography (DSA), which included the vertebral artery V4, proximal basilar artery, middle basilar artery, and distal basilar artery. The mTICI score assessed on the final angiogram indicated successful reperfusion, which was graded as 2b or 3 (4). Perioperative complications, including symptomatic intracranial hemorrhage (sICH), thromboembolism, thrombosis or plaque shedding, arterial dissection and vessel perforation, and patency at 24–72 h on computed tomography angiography (CTA) or magnetic resonance angiography (MRA) were also recorded.

### 2.1 Ethics approval

Ethical review and approval was not required for the study on human participants in accordance with the local legislation and institutional requirements. Written informed consent from the patients/participants or patients/participants' legal guardian/next of kin was not required to participate in this study in accordance with the national legislation and the institutional requirements.

### 2.2 Statistical analysis

Clinical and radiological outcomes were dichotomized into the favorable recanalization group (defined as a 90-day mRS score ≤ 3) and the futile recanalization group (defined as a 90-day mRS score > 3). The association between futile recanalization and baseline clinical variables, imaging characteristics, and acute stroke workflow interval times was examined in the univariate analysis. Continuous data were summarized as a median and interquartile range (IQR) or as means and standard deviation. Means and medians were compared using the *t*-test and Mann–Whitney *U*-test, respectively. Frequencies were compared using the *χ^2^* test. Multivariate logistic regression was used to determine the relationship between confounders and futile recanalization. To maximize sensitivity, variables with a *p*-value of <0.05 were entered into the multivariate logistic regression with a backward likelihood ratio model. The confounders adjusted for included age, diabetes mellitus, baseline NIHSS score, and baseline GCS score. Data are presented as the adjusted odds ratio (OR) and its 95% confidence interval (95% CI). All analyses were performed using SPSS software, version 22.0 (SPSS, Chicago, IL, USA), with a significance level of a *p*-value of <0.05 (two-sided).

## 3 Results

**Figure 1:**
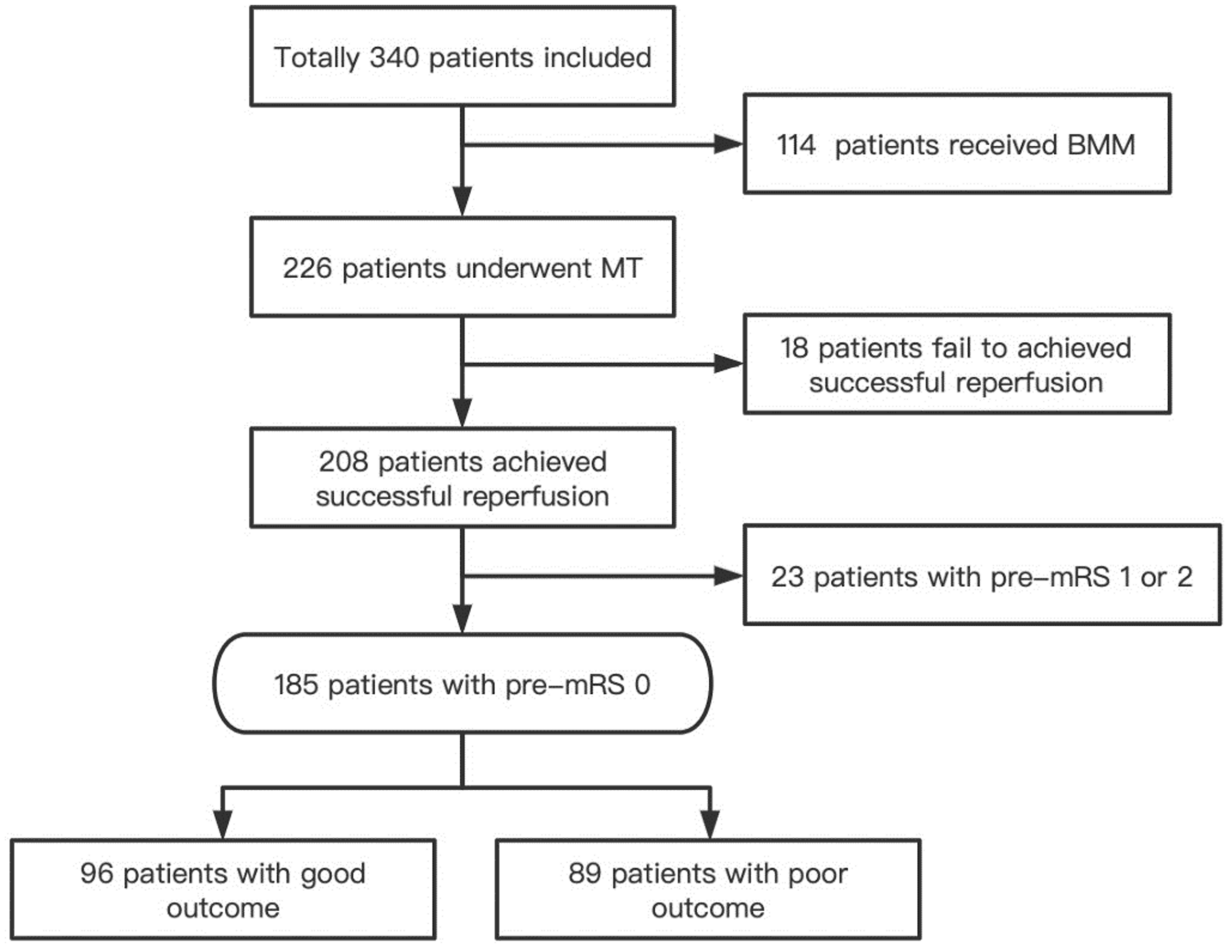
Flowchart. BMM, Indicate best medical management; MT, Mechanical thrombectomy; mRS, Modified rankin scale.

From 21 February 2021 to 3 January 2022, a total of 507 patients were assessed for eligibility for the ATTENTION trial; a total of 342 patients were enrolled in the trial (4). In total, 228 patients were allocated to the thrombectomy group and 114 were allocated to the best medical treatment. The legal representatives of two patients who had been assigned to the thrombectomy group withdrew their consent, so 226 patients in the thrombectomy group were included. Successful reperfusion was achieved in 208 (92.0%) patients. After excluding 23 patients with pre-mRS scores of 1 or 2, 185 patients were included in the analysis (Figure 1).

Futile recanalization was observed in 89 (48.1%) patients, favorable recanalization in 96 (51.9%) patients, and death in 57 (30.8%) patients. The baseline characteristics of the patients are listed in Table 1. Compared with the patients in the favorable recanalization group, the patients in the futile recanalization group were significantly older (mean 69 vs. 63 years, *p* = 0.001) and had significantly higher NIHSS scores (median 35 vs. 18, *p* < 0.001) on admission, lower admission baseline GCS scores (median 6 vs. 10, *p* < 0.001), and a higher prevalence of diabetes mellitus (25.8% vs. 13.5%, *p* = 0.035).

**Table 1:** Baseline characteristics of patients with futile and favorable recanalizations.

	All patients (*n* = 185)	Favorable recanalization (*n* = 96)	Futile recanalization (*n* = 89)	*p*-value
Age (year), mean	66 ± 11.3	63. ± 11.6	69 ± 11.6	0.001
Baseline NIHSS score, median (IQR)	26 (15–35)	18 (13–33)	35 (22–37)	<0.001
Baseline GCS score, median (IQR)	8 (6–12)	10 (7–13)	6 (4.5–9)	<0.001
Female sex, n (%)	60 (32.4)	31 (32.3)	29 (32.6)	0.966
Risk factors, *n* (%)				
Hypertension	127 (68.6)	69 (71.9)	58 (65.2)	0.326
Hyperlipidemia	47 (25.4)	23 (24.0)	24 (27.0)	0.639
Diabetes mellitus	36 (19.5)	13 (13.5)	23 (25.8)	0.035
Atrial fibrillation	40 (21.6)	19 (19.8)	21 (23.6)	0.530
Smoking	55 (29.7)	30 (31.3)	25 (28.1)	0.638
Coronary artery disease	29 (15.7)	12 (12.5)	17 (19.1)	0.217
Transient ischemic attack and ischemic stroke	32 (17.3)	17 (17.7)	15 (16.9)	0.878
SBP, median (IQR)	150 (133.5–168.5)	149 (131.25–165)	150 (135–171)	0.231
DBP, median (IQR)	85 (77–96.5)	83 (76.25–94)	87 (77.5–100)	0.122
Stroke cause, *n (%)				
Large-artery atherosclerosis	88 (47.6)	49 (51.0)	39 (43.8)	0.359
Intracranial artery -LAA	73 (39.5)	43 (44.8)	30 (33.7)	
Extracranial artery-LAA	15 (8.1)	6 (6.3)	9 (10.1)	
Cardioembolism	41 (22.2)	23 (24.0)	18 (20.2)	
Undetermined cause	54 (29.2)	23 (24.0)	31 (34.8)	
Other determined cause	2 (1.1)	1 (1.0)	1 (1.1)	
Intravenous thrombolysis, *n* (%)	57 (30.8)	33 (34.4)	24 (27.0)	0.276
Occlusion site, *n* (%)				
Vertebral artery V4	16 (8.6)	10 (10.4)	6 (6.7)	0.769
Proximal basilar artery	54 (29.2)	27 (28.1)	27 (30.3)	
Middle basilar artery	48 (25.9)	25 (26.0)	23 (25.8)	
Distal basilar artery	66 (35.7)	33 (34.4)	33 (37.1)	
pc-ASPECT, median (IQR)	9 (8–10)	9 (8–10)	9 (8–10)	0.936
General anesthesia, *n* (%)	128 (69.2)	65 (67.7)	63 (70.8)	0.650
Onset-to-puncture, median (h), median (IQR)	5.61 (3.53–7.48)	5.64 (3.46–7.31)	5.57 (3.73–7.85)	0.573
Time-to-treatment ≤6/>6 h, *n* (%)				
0–6 h	105 (56.8)	54 (56.3)	51 (57.3)	0.885
6–12 h	80 (43.2)	42 (43.8)	38 (42.7)	
Onset-to-reperfusion time, median (*h*)	6.84 (4.82–8.74)	6.72 (4.72–8.46)	7.25 (5.26–8.87)	0.325
First pass effect, *n* (%)	110 (59.5)	61 (63.5)	49 (55.1)	0.240
Complication during procedure, *n* (%)				
Thromboembolism	17 (9.2)	7 (7.3)	10 (11.2)	0.394
Acute occlusion	1 (0.5)	1 (1.0)	0	
Thrombosis or plaque shedding:	1 (0.5)	0	1 (1.1)	
Dissection	6 (3.2)	3 (3.1)	3 (3.4)	
Vessel perforation	2 (1.1)	0	2 (2.2)	
Successful recanalization, *n* (%)				0.419
TICI 3 post-operation	146 (78.9)	78 (81.2)	68 (76.4)	
TICI 2b post-operation	39 (21.1)	18 (18.7)	21 (24.6)	
AOL post-operation, *n* (%)				
0	1/142 (0.7)	1/94 (1.1)	0/48 (0)	0.885
1	9/142 (6.3)	6/94 (6.4)	3/48 (6.3)	
2	27/142 (19.0)	17/94 (18.1)	10/48 (20.8)	
3	105/142 (73.9)	70/94 (74.5)	35/48 (72.9)	
ICH at 24–72 h as assessed radiologically, *n* (%)	8 (4.3)	2 (2.1)	6 (6.7)	0.157
sICH according to SITS-MOST criteria at 24–72 h, *n* (%)	8 (4.3)	2 (2.1)	6 (6.7)	0.157
Any ICH according to SITS-MOST criteria at 24–72 h, *n* (%)	23 (12.4)	8 (8.3)	15 (16.9)	0.079
Severe adverse event, *n* (%) AE				
HF	7 (3.8)	3 (3.1)	4 (4.5)	0.712
RF	2 (1.1)	0	2 (2.2)	0.230
LF	1 (0.5)	0	1 (1.1)	0.481
Anemia	7 (3.8)	3 (3.1)	4 (4.5)	0.712
Gastrointestinal bleeding	26 (14.1)	14 (14.6)	13 (13.5)	1.000
Severe pneumonia	9 (4.9)	3 (3.1)	6 (6.7)	0.316

NIHSS, National Institutes of Health Stroke Scale; IQR, interquartile range; GCS, Glasgow coma scale; SBP, systolic blood pressure; DBP, diastolic blood pressure; LAA, large-artery atherosclerosis; pc-ASPECT, posterior circulation alberta stroke program early ct score, TICI, thrombolysis in cerebral infarction; AOL, arterial occlusive lesion; ICH, intracranial hemorrhage; sICH, symptomatic intracranial hemorrhage; AE, adverse event; HF, heart failure; RF, renal failure; LF, liver failure.

**Figure 2:**
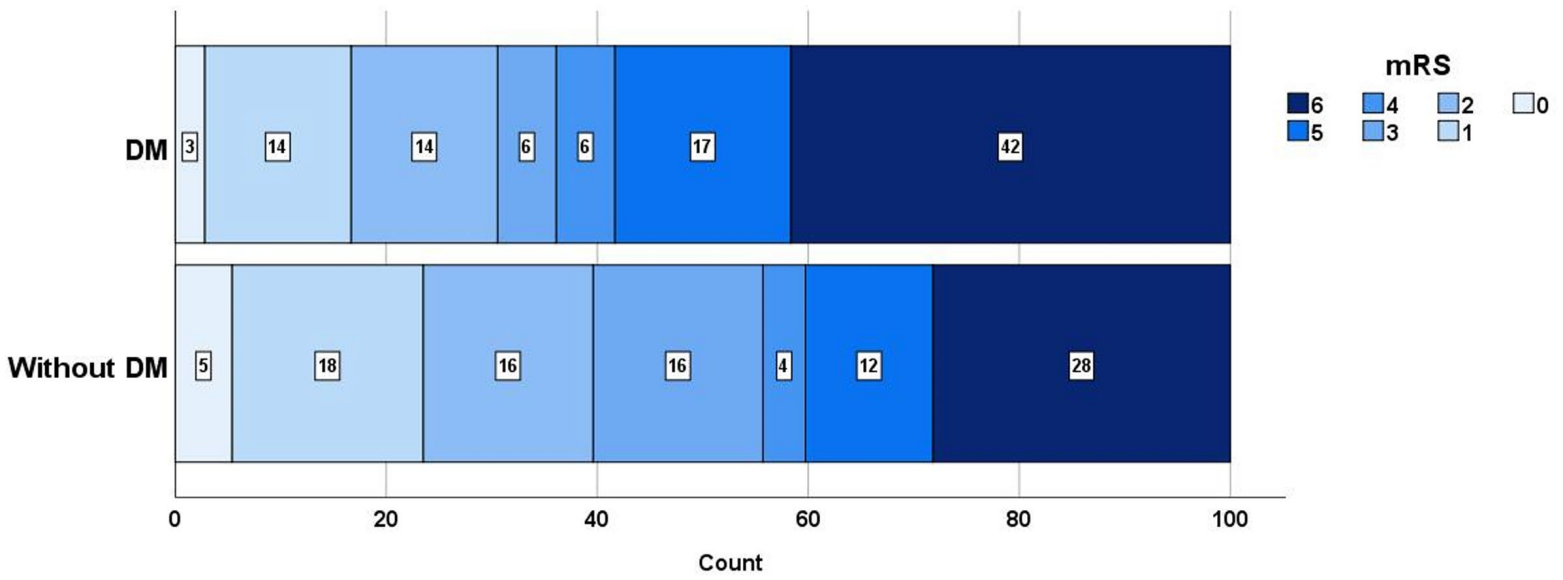
mRS distribution in patients with DM and without DM. mRS, Indicate modified Rankin scale; DM, Diabetes mellitus.

There were 36 patients with diabetes mellitus and 149 patients without diabetes mellitus. Patients with diabetes had a higher mortality rate (25.0% vs. 20.1%, *p* = 0.521), a higher prevalence of sICH (8.8% vs. 3.6%, *p* = 0.196), and less functional independence (36.1% vs. 55.7%, *p* = 0.035) than those without diabetes (Figure 2). Patients with TICI 3 reperfusion had a trend of a lower mortality rate (30.1% vs. 33.3%, *p* = 0.701) and a better prognosis (53.4% vs. 46.2%, *p* = 0.419) than patients with TICI 2b reperfusion.

In the multivariable logistic regression analysis (Table 2), the following factors were associated with futile recanalization: older age (OR 1.04, 95% CI 1.01–1.08, *p* = 0.01) and diabetes mellitus (OR 3.35, 95% CI 1.40–8.01, *p* = 0.0107).

**Table 2:** Logistic regression analysis identifying independent predictors of futile recanalization.

Factor	Univariate		Multivariate	
	OR (95% CI)	*p*	OR (95% CI)	*p*
Age, per 1-year increase	1.05 (1.02–1.08)	0.002	1.04 (1.01–1.08)	0.010
Baseline NIHSS score, per 1-point increase	1.09 (1.06–1.12)	<0.001	1.04 (0.98–1.11)	0.175
Baseline GCS score, per 1-point increase	0.79 (0.72–0.86)	<0.001	0.85 (0.72–1.01)	0.062
Diabetes mellitus, yes	2.23 (1.05–4.72)	0.037	3.35 (1.40–8.01)	0.007

NIHSS, Indicate National Institutes of Health Stroke Scale; GCS, Glasgow coma scale.

## 4 Discussion

Due to the development of EVT techniques and adjustment of patient selection (i.e., NIHSS>6), trials have shown that patients with acute ischemic stroke caused by basilar artery occlusion can benefit from EVT (4, 5). However, despite successful recanalization, some patients suffer a poor prognosis, a condition referred to as futile recanalization. In the current study, a subgroup analysis from the ATTENTION research revealed that the rate of futile recanalization was 48.1%, which is similar to the 45.1% (6) and 46.9% (7) reported previously but lower than the 62.8% (8). The highest rate was reported in the study by Yang et al. (8), and the main reason may be the different onset-to-reperfusion times, in which presentation within 24 h was included in Yang et al.’s study, and only presentation within 12 h was included in our study and 8 h in Pop et al.’s study.

Our post-hoc analysis demonstrated that old age and diabetes mellitus were associated with a higher risk of futile recanalization. Consistent with a previous study, older age was associated with futile recanalization (6, 9)in acute BAO patients, which is similar to the results observed in acute anterior circulation LVO (10). Age is a common factor that is associated with clinical outcomes in patients with LVO who underwent EVT. The HERMES meta-analysis showed that in patients over 80 years old who received EVT, the good prognosis rate decreased to 29.8% (10), and the functional independence rate decreased with increasing age in patients with low ASPECTS (11). The possible reasons are listed as follows: a. Elderly patients often have a poor arterial collateral network (9), which is associated with a good prognosis; b. Elderly patients are more likely to have cardiovascular risk factors, such as hypertension, diabetes mellitus, and smoking, which can impair endothelial function and stiffen the vessels’ myogenic tone, affecting the self-regulating ability of arteries (12); c. Leukoaraiosis is more common in elderly patients and is an imaging marker of poor prognosis in patients who receive EVT (13); d. Elderly patients often have more comorbidities that limit their complete rehabilitation.

Our study revealed that diabetes mellitus is another factor that is associated with futile recanalization. A meta-analysis has demonstrated that diabetes mellitus is linked to unfavorable functional outcomes, increased mortality, and poor postprocedural safety outcomes, including symptomatic intracranial hemorrhage (sICH) and hypertension (HT) (14). Genceviciute also discovered that diabetes mellitus, especially admission hyperglycemia, was associated with less frequently successful reperfusion, worse 3-month functional outcomes, and in-hospital symptomatic intracranial hemorrhage in acute anterior circulation stroke patients who underwent EVT (15, 16). The possible reason for these findings may be that hyperglycemia is associated with an increased risk of infarct growth as it may potentially increase the vulnerability of the penumbra (15), and hyperglycemia is associated with unsuccessful recanalization. An animal study showed increased MMP-9, the receptor for advanced glycation end products, and vascular endothelial growth factor in diabetic mice, which were associated with increased blood–brain barrier leakage (17), which can explain why hemorrhage transformation was more commonly observed in acute stroke patients with diabetes mellitus who received reperfusion therapy than in patients without diabetes mellitus. Consistent with a previous study, our study showed that sICH was more common in patients with diabetes mellitus although without statistical significance probably due to the small sample size.

Compared with incomplete reperfusion, complete reperfusion (18), especially the first pass to achieve complete reperfusion, which is called the first pass effect (19–21), is related to better outcomes. De Havenon et al.’s study also showed that the benefit of recanalization in BAO patients was significantly decreased when recanalization was achieved after more than three attempts (22). However, in our study, we did not observe a correlation between the first-pass effect and clinical outcome, and this phenomenon was also observed in another study (6, 8). Interestingly, Yang’s study found that stent retriever passes were associated with futile recanalization in patients with late time windows (defined as 6–24 h), but the association was not observed in the early time window group (8). The discrepancy between the two groups may be attributed to the following reasons: the scope of the ischemic penumbra was larger in the early time window than in the late time window, which may minimize the influence of mechanical thrombectomy passes, and this can also explain the results of our study for the median onset-to-puncture’s time was 5.61 h, which was obviously shorter than that in Yang et al.’s study (8.88 h) (8).

Our study did not find any specific futile recanalization predictive factors that were not reported in previous studies. However, there were some strengths of our study: first, the dataset was acquired through a nationwide multicentric randomized controlled trial of consecutive thrombectomy procedures, and second, a systematic independent 90-day follow-up with adjudication of clinical outcomes was performed. All these factors make the data more reliable than that in the registry study. Nevertheless, there are certain limitations of our study. First, although data were prospectively registered, all data of this subgroup analysis were retrospectively assessed. Second, patients with a prestroke mRS score of ≥1 were excluded to reduce the influence of a previous stroke history on the clinical outcome. Third, the sample size is not large enough to explore more variables that are associated with futile recanalization. Fourth, blood glucose was not recorded on admission, which is a better indicator of futile recanalization. Fifth, the term futile recanalization suggests that patients might as well not have undergone mechanical thrombectomy; however, our study did not compare the therapeutic effect of mechanical thrombectomy with the best medical management, so we cannot know what the outcome of these patients would have been without endovascular treatment.

## 5 Conclusion

In our post-hoc analysis, we observed that futile recanalization is common in patients with BAO following endovascular treatment. Old age and diabetes mellitus were independent predictors of futile recanalization after endovascular therapy for acute BAO.

## Data availability statement

The original contributions presented in the study are included in the article/supplementary material, further inquiries can be directed to the corresponding author.

## Ethics statement

Written informed consent was obtained from the individual(s) for the publication of any potentially identifiable images or data included in this article.

## Author contributions

TY: Writing – original draft, Writing – review & editing, Conceptualization, Formal analysis, Funding acquisition. KL: Conceptualization, Data curation. X-hL: Data curation, Methodology. D-lL: Data curation, Formal analysis. Y-MW: Data curation, Methodology. Z-nP: Data curation, Methodology. X-fZ: Data curation, Methodology. R-cC: Data curation, Methodology. GZ: Data curation, Methodology. W-hC: Conceptualization, Methodology, Supervision, Validation, Writing – review & editing.
